# Which diet has the lower water footprint in Mediterranean countries?

**DOI:** 10.1016/j.resconrec.2021.105631

**Published:** 2021-08

**Authors:** Davy Vanham, Susann Guenther, Marta Ros-Baró, Anna Bach-Faig

**Affiliations:** a European Commission, Joint Research Centre (JRC), Ispra, Italy; b Arcadia SIT S.r.l., 27029 Vigevano, Italy; c FoodLab Research Group (2017SGR 83), Faculty of Health Sciences, Universitat Oberta de Catalunya (Open University of Catalonia, UOC), 08018 Barcelona, Spain; d Food and Nutrition Area, Barcelona Official College of Pharmacists, 08009 Barcelona, Spain

**Keywords:** Water footprint, Sustainable diet, Mediterranean diet, EAT-Lancet diet, Water resources, Food system

## Abstract

The Mediterranean region is increasingly water scarce, with the food system being the largest driver of water use. We calculate the water resources related to food consumption in nine major Mediterranean countries, by means of the water footprint (WF), for the existing situation (period 2011-2013) as well as the Mediterranean and EAT-Lancet diets. We account for different food intake requirements according to gender and six age groups. These nine countries – Spain, France, Italy, Greece, Turkey, Egypt, Tunisia, Algeria and Morocco - represent 88% of the population of all countries bordering the Mediterranean. As first major observation, we find that the EAT-Lancet diet, a scientifically optimised diet for both nutrition and certain environmental indicators, requires less water resources than the Mediterranean diet, a culturally accepted diet within the region. In terms of water resources use, adherence to the former is thus more beneficial than adherence to the latter. As second major observation, we find that the EAT-Lancet diet reduces the current WF for all nations consistently, within the range -17% to -48%, whereas the Mediterranean diet reduces the WF of the European countries, Turkey, Egypt and Morocco within the range of -4% to -35%. For the Maghreb countries Tunisia and Algeria, the Mediterranean diet WF is slightly higher compared to the current WF and the proportions of food product groups differ. Such dietary shifts would be important parts of the solution to obtain the sustainable use of water resources in Mediterranean countries.

## Graphical Abstract

**Figure:**
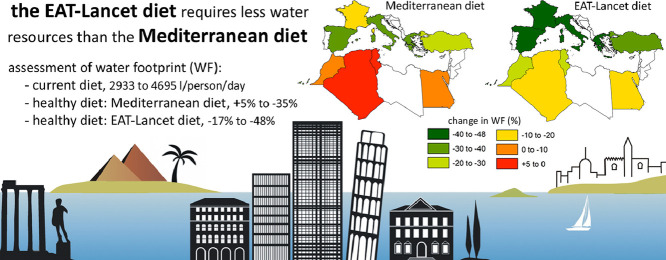

## 1 Introduction

Many people in the Mediterranean region already face moderate to high water stress, especially during summer months (Mekonnen and Hoekstra, 2016). Modelling studies have shown that dietary changes are required to improve the health of humanity while at the same time keeping the food system within planetary boundaries, including the boundary for water (Springmann et al., 2018; Willett et al., 2019). Achieving both the United Nations Sustainable Development Goal (SDG) 2 on food security and SDG 6 on water security in a water-energy-food-ecosystem nexus (Vanham et al., 2019) context requires shifts to diets that are both nutritious and sustainable (FAO and WHO, 2019). Applying such an integrated nexus approach, crossing disciplinary and institutional borders, is a prerequisite, as the food and water sectors are intrinsically linked (Bleischwitz et al., 2018; Liu et al., 2018; Markantonis et al., 2019; Vanham, 2016).

In economic terms, annually, the world food system generates about $ 10 trillion, but it costs about $ 12 trillion in poor health and ecological damage (Nature editorial, 2019). Growing overweight and obesity among adults and children (Abarca-Gómez et al., 2017) is a manifestation of poor health conditions. Also in Mediterranean countries an increase in overweight and obesity is observed (Abarca-Gómez et al., 2017; Atek et al., 2013; Cattaneo et al., 2010; Galal, 2006; Nasreddine et al., 2018; Prosperi et al., 2014). On a global level, a transition to plant-based diets is estimated to cost $ 30 billion, but the resulting economic benefits are predicted to be around $ 1.28 trillion (Nature editorial, 2019). Water is essential for human health, all economic sectors as well as the environment. Global economic losses from inadequate water supply and sanitation amount to $ 260 billion per year, whereas water insecurity to existing irrigators amounts to $ 94 billion per year in losses (Sadoff et al., 2015). Further, water-related losses in agriculture, health, income, and property could result in a decline by as much as 6% of GDP by 2050 in some regions of the world and spur sustained negative growth (World Bank, 2016). Sustainable development towards healthy diets can thus also provide economic benefits.

A recently published FAO and WHO report (FAO and WHO, 2019) defines sustainable healthy diets as “*dietary patterns that promote all dimensions of individuals’ health and wellbeing; have low environmental pressure and impact; are accessible, affordable, safe and equitable; and are culturally acceptable*”. Diets that have been discussed as both healthy and environmentally sustainable, include the Mediterranean diet (Bach-Faig et al., 2011; Hachem et al., 2020; Schröder et al., 2004; Tilman and Clark, 2014) and the EAT-Lancet reference diet (Willett et al., 2019). The Mediterranean diet is a territorial diet that has its roots entrenched in the history of the Mediterranean Sea and its region (Hachem et al., 2020). The traditional Mediterranean diet was defined originally as a diet with high consumption of whole cereals, legumes, vegetables, fruits, nuts and olive oil, a low to mild consumption of dairy products, and a low consumption of meat and poultry. The Mediterranean Diet is declared as an intangible cultural heritage by UNESCO in 2010 (UNESCO, 2013) and is much promoted by institutions such as the FAO (CIHEAM/FAO, 2015). While the Mediterranean Diet is a culturally acceptable model in the Mediterranean basin, this is not the case for the EAT-Lancet reference diet, as latter is a new scientifically optimised diet for both nutrition and certain environmental indicators.

Due to freshwater use in different economic activities including agriculture, both blue and green water resources are considered scarce (Hoekstra and Wiedmann, 2014; Schyns et al., 2019). Blue water refers to water in rivers, lakes and aquifers. Green water is the soil water held in the unsaturated zone, formed by precipitation and available to plants (Falkenmark et al., 2019). Rainfed agriculture receives only green water while irrigated agriculture receives blue water (from irrigation) as well as green water (from precipitation). The water footprint is an environmental footprint that measures consumptive green and blue water use along a supply chain (Hoekstra and Mekonnen, 2012; Vanham et al., 2019), thereby linking water resources to food consumption. Food consumption generally makes up the largest proportion in an individual's total WF, far exceeding the amounts of water used at home (Hoekstra and Mekonnen, 2012; Jalava et al., 2014; Kassem et al., 2021). Water footprint analyses, including related to different diets, have been conducted for certain Mediterranean countries (Abdelkader et al., 2018; Blas et al., 2019; Chouchane et al., 2015; Kim et al., 2019; Schyns and Hoekstra, 2014) and cities (Vanham et al., 2016). Here we analyse the water footprint of food consumption in nine Mediterranean countries for the existing situation (REF, 2011-2013) as well as two diet scenarios, i.e the Mediterranean (MEDIT) and EAT-Lancet reference (EAT-LANCET) diets. We thus account for the pressure water resource use, not its impact water stress (Vanham, 2020; Vanham and Leip, 2020; Vanham and Mekonnen, 2021). We use recommended food product group intake amounts from Bach-Faig et al. (2011) for MEDIT and from Willett et al. (2019) for EAT-LANCET. In order to formulate the dietary scenarios, we account for different food intake requirements according to gender and six age groups. For male adults the target energy intake is set at 2500 kcal/day, for female adults at 2000 kcal/day. Our analysis is novel. The comprehensive assessment of nine countries, accounting for 88% of the population bordering the Mediterranean, including the Mediterranean and EAT-Lancet diet scenarios and accounting for gender and age-specific food intake recommendations, has never been done before. For the first time, we thereby also compare water resources requirements for the Mediterranean and EAT-Lancet diet, identifying which diet is the most water efficient.

## 2 Methods

### 2.1 Current food intake and diet scenarios

We use average annual FAO Food Balance Sheets (FBS)(FAO, 2001; FAOSTAT, 2019) food supply data for the period 2011-2013 (the most recent data available when we conducted our analysis) to compute current (REF) food intake data. We use the food product groups as defined in the FAO FBS. To obtain national food intake data from these food supply data, two correction factors are used. The first accounts for product primary equivalent conversion (because FAO FBS food supply data are provided in primary equivalents), and the second for consumer food waste. This approach is described in detail in Vanham et al. (2013). For the consumer food waste factor, we use average EU data (Vanham et al., 2015) for the European countries and FAO data (Gustavsson et al., 2011) for Turkey and the North African countries. The REF food supply energy amounts, provided in the FAO FBS, as well as calculated REF energy intake amounts are listed in Table 1. Both values represent the whole national population.

**Table 1:** Total energy amounts (kcal/day) per country, for REF (actual current population weighted food supply and intake amounts) as well as for the 2 diet scenarios. All values without stimulants, alcoholic beverages and spices

Country	REF		MEDIT		EAT-LANCET		Population weighted target energy for the 2 diet scenarios
	FAO food supply, total population	FAO food intake, total population	Adult Male	Adult Female	Adult Male	Adult Female	
Algeria	3236	2955	2500	2000	2500	2000	2009
Egypt	3490	3155	2500	2000	2500	2000	1979
France	3307	2897	2500	2000	2500	2000	2021
Greece	3287	2872	2500	2000	2500	2000	2039
Italy	3452	3015	2500	2000	2500	2000	2037
Morocco	3339	3030	2500	2000	2500	2000	2007
Spain	2994	2632	2500	2000	2500	2000	2048
Tunisia	3321	3037	2500	2000	2500	2000	2036
Turkey	3705	3410	2500	2000	2500	2000	2016

**Fig. 1:**
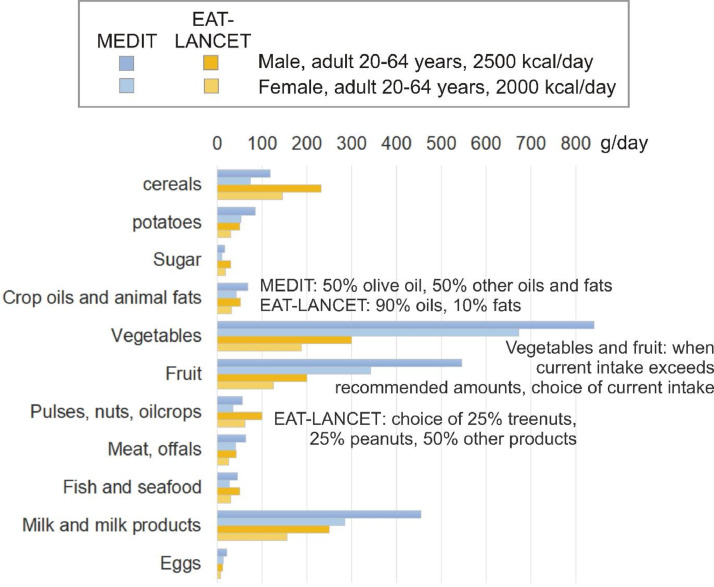
Recommended intake amounts (gram per day) for each product group for the MEDIT and EAT-LANCET diets for male and female adults (age group 20-64 years old), based on (Bach-Faig et al., 2011) and (Willett et al., 2019)

We analyse two diet scenarios: the Mediterranean diet (MEDIT) as defined by Bach-Faig et al. (2011) and the EAT-Lancet reference diet (Willett et al., 2019) (EAT-LANCET). We differentiate recommended food product group intake amounts according to gender (male and female) and six age groups (0 to 4, 5 to 9, 10 to 14, 15 to 19, 20 to 64 years old and 65 and older). For male adults the target energy intake is set at 2500 kcal/day, for female adults at 2000 kcal/day. According to nutritional recommendations, other age groups have different target energy intake amounts. Comparing the environmental pressures and impacts of dietary patterns in an isocaloric manner is a common procedure in many studies (Batlle-Bayer et al., 2020; Blas et al., 2019; Blas et al., 2016; Heller et al., 2013; Kassem et al., 2021). Fig. 1 shows the recommended intake amounts per product group (in gram per day) for male and female adults, based on the chosen target energy intake amounts. The amounts per age group are listed in Table 2. For the food product groups stimulants and spices, the current intake amounts are kept constant. For alcoholic beverages, we use WHO recommendations of maximum 20 g/day pure alcohol for men (2 standard drinks) and maximum 10 g/day pure alcohol for women (1 standard drink). We use these amounts for adults (+18 year olds). Up to 18 years, zero alcohol intake is set. Population statistics for each country were retrieved from the UN population databases (UN, 2020). For the food product groups stimulants and spices, we maintain REF intake amounts for the diet scenarios.

**Table 2:** Recommended intake amounts (gram per day) for each product group for the MEDIT and EAT-LANCET diets, according to gender and age group

Product group	Age group	MEDIT		EAT-LANCET	
		Male	Female	Male	Female
cereals	0 to 4	72	57	139	111
	5 to 9	95	76	186	148
	10 to 14	119	95	232	186
	15 to 19	156	108	302	210
	20 to 64	119	75	232	146
	65 and older	95	76	186	148
potatoes	0 to 4	51	41	30	24
	5 to 9	68	55	40	32
	10 to 14	85	68	50	40
	15 to 19	111	77	65	45
	20 to 64	85	54	50	31
	65 and older	68	55	40	32
sugar	0 to 4	10	8	19	15
	5 to 9	14	11	25	20
	10 to 14	17	14	31	25
	15 to 19	23	15	40	28
	20 to 64	17	11	31	19
	65 and older	14	11	25	20
Crop oils and animal fats	0 to 4	41	33	31	25
	5 to 9	55	44	41	33
	10 to 14	68	55	52	41
	15 to 19	89	62	67	47
	20 to 64	68	44	52	33
	65 and older	55	44	41	33
vegetables	0 to 4	505	404	180	144
	5 to 9	673	538	240	192
	10 to 14	841	673	300	240
	15 to 19	1093	875	390	271
	20 to 64	841	673	300	188
	65 and older	673	538	240	192
fruit	0 to 4	327	262	120	96
	5 to 9	436	349	160	128
	10 to 14	545	436	200	160
	15 to 19	709	494	260	181
	20 to 64	545	343	200	126
	65 and older	436	349	160	128
Pulses, nuts, oilcrops	0 to 4	34	27	20	16
	5 to 9	45	36	27	21
	10 to 14	57	45	33	27
	15 to 19	74	51	43	30
	20 to 64	57	36	100	63
	65 and older	45	36	27	21
Meat, offals	0 to 4	39	31	26	21
	5 to 9	52	42	34	27
	10 to 14	65	52	43	34
	15 to 19	84	59	56	39
	20 to 64	65	41	43	27
	65 and older	52	42	34	28
Fish, seafood	0 to 4	27	22	36	29
	5 to 9	36	29	40	32
	10 to 14	45	36	50	40
	15 to 19	59	41	65	45
	20 to 64	45	28	50	31
	65 and older	36	29	40	32
milk	0 to 4	273	218	150	120
	5 to 9	364	291	200	160
	10 to 14	455	364	250	200
	15 to 19	591	411	325	226
	20 to 64	455	285	250	157
	65 and older	364	291	200	160
eggs	0 to 4	13	10	8	6
	5 to 9	17	14	10	8
	10 to 14	22	17	13	10
	15 to 19	27	19	17	12
	20 to 64	22	14	13	8
	65 and older	17	14	10	8

To respect national food intake preferences, we use the same mass proportion of products within a product group for the diet scenarios as for REF. As an example, when dates represent a proportion of 30% within “fruit” in REF, also in the recommended “fruit” amount for MEDIT and EAT-LANCET they represent 30%. Only in certain food product groups there are exceptions to this rule, when specifically defined as such in the diet specifications (Fig. 1). In the food product group “crop oils and animal fats”, for MEDIT 50% is olive oil, the other 50% remaining oils and animal fats. For EAT-LANCET, 90% are oils (proportions according to current preference) and 10% animal fats. In the product group “pulses, nuts, oilcrops”, for EAT-LANCET, 25% are treenuts, 25% peanuts and 50% remaining products (proportions according to current preference). For MEDIT, the general rule on proportion is followed.

For the product groups “fruit” and “vegetables”, when the REF intake exceeds recommended MEDIT or EAT-LANCET amounts, the REF intake is chosen for the diet scenarios (Fig. 1), as fruits and vegetables are healthy products which do not require an upper limit.

### 2.2 The water footprint (WF) of food consumption

The consumptive water footprint (WF) accounts for green and blue water resources. Here we compute for each country a national WF of consumption based upon the FAO FBSs (average annual amounts for 2011-2013) and respective unit WF amounts (m^3^/ton) as listed in the international WF database for crops and crop products of Mekonnen and Hoekstra (2011) as well as for livestock products of Mekonnen and Hoekstra (2012).

For each of the food items in the FAO FBS (N=80), we calculated the WF of national consumption. These amounts result from the WF of production of domestically produced and imported products, according to the same proportion of domestic production and import to the total domestic supply in the FAO FBS. For each food item, we take average annual import data for the period 2011-2013 from FAOSTAT (2019). For the import, we quantify the WF of consumption based upon the unit WF of production in importing countries, to a minimum of 50% of total import quantity. For the remaining percentage, we use the global average. As an example, for Morocco, 61% of the domestic wheat supply comes from domestic production and 39% from import. Of import quantities, the main countries of origin are France (25%), Canada (16%) and Argentina (15%), combined responsible for 56% of imports. The WF for 178 kg/person/year wheat consumption is then calculated for 61% with the national unit WF of production (2758 m^3^/ton green and 245 m^3^/ton blue) and for 39% by import. Latter value is calculated for 25% with the national WF of France (581 m^3^/ton green and 1 m^3^/ton blue), 16% Canada (1336 m^3^/ton green and 5 m^3^/ton blue), 15% Argentina (1770 m^3^/ton green and 11 m^3^/ton blue) and the remaining 44% global average (1277 m^3^/ton green and 342 m^3^/ton blue). The resulting green and blue WF of Moroccan wheat consumption then amount to 1049 and 102 l/person/day respectively. We also include a WF for aquaculture fish and seafood, based on Pahlow et al. (2015).

For the diet scenarios, we calculate an average food product intake amount weighted according to different population groups (as listed in Table 2) and related national population statistics. Latter gender and age population statistics we retrieve from UN population databases (UN, 2020). The WF of consumption per food group then increases or decreases with respect to REF, according to these dietary intake recommendations.

### 2.3 Water efficiency of olive oil and fruit in Maghreb countries

**Fig. 2:**
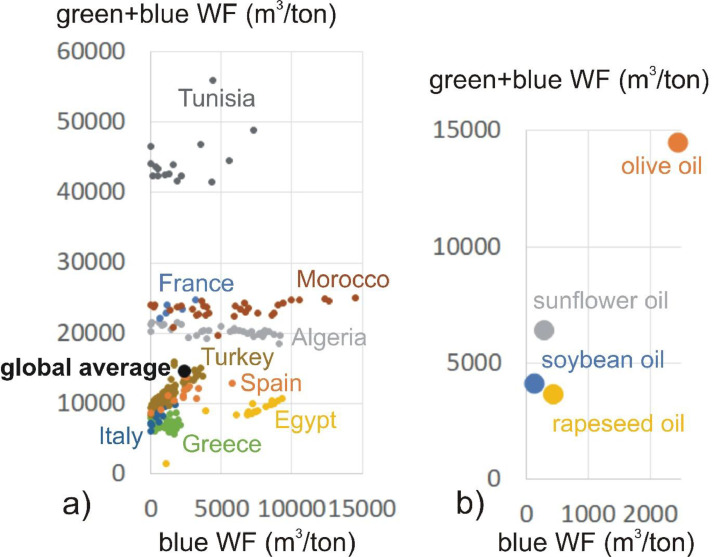
The green plus blue as well as blue unit WF of production (in m^3^/ton) of a) olive oil in different regions within the nine countries and b) different oil types as global average. Data source (Mekonnen and Hoekstra, 2011)

One of the three points for a full sustainability assessment of a dietary WF (Vanham, 2020) is an efficiency assessment for each food item in the diet. We do not conduct this for all food items, but for a selection of products that are strategically important within the diets of Mediterranean countries. In the Maghreb countries, many food products that contribute large proportions to the total WF of a diet, are produced in a water inefficient way. This means they have large unit WF of production amounts (m^3^/ton) as compared to the global average or a set WF benchmark (Mekonnen and Hoekstra, 2014). As an example, Fig. 2 shows the large range in the green plus blue (1527-23463 m^3^/ton) as well as the blue (0-15007 m^3^/ton) unit WF of production of olive oil in subnational areas of the nine Mediterranean countries. The global average amount is 14504 m^3^/ton for the green plus blue WF and 2437 m^3^/ton for the blue WF.

We find that olive oil and fruit have large proportions in the total REF, MEDIT and EAT-LANCET WF in the Maghreb countries. Therefore, we conduct in these countries an additional scenario for each diet by assuming all olive oil as well as the most consumed fruit (oranges, apples, grapes, dates) produced with a global average unit WF.

## 3 Results

**Fig. 3:**
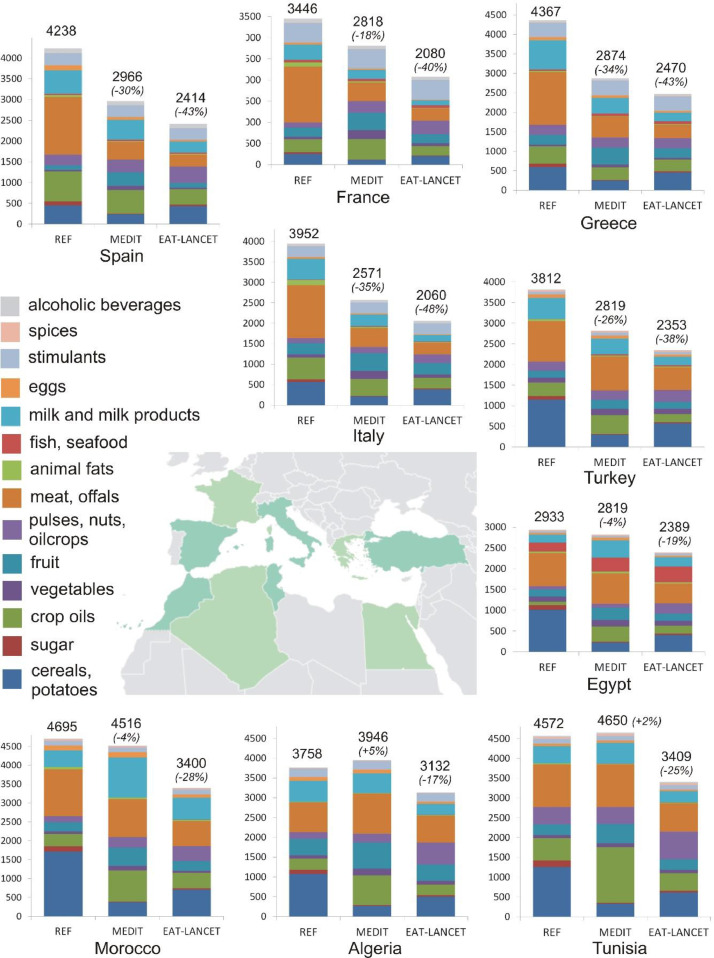
The green plus blue WF of consumption (in litres per person per day or l/person/day) for the nine countries, for REF, MEDIT and EAT-LANCET.

The total REF (green+blue) WF of consumption ranges from 2933 l/person/day (Egypt) to 4695 l/person/day (Morocco), with a median amount of 3952 l/person/day (Italy)(Fig. 3). The Maghreb countries show the lowest proportion of animal products to these total WF amounts (Morocco 40%, Algeria 37% and Tunisia 35%). In Egypt and Turkey these proportions are 44% respectively 43%. In the European countries the proportion of animal products accounts for half or more of the total WF amount (Greece 52%, Italy 50%, France 55% and Spain 51%).

A shift to a MEDIT diet decreases the total WF of consumption in European countries as well as Turkey substantially, within the range of -18% to -35% (France -18%, Turkey -26%, Spain -30%, Greece -34% and Italy -35%). The largest fraction of this decrease is accountable to less meat intake (Table 3). Product groups for which the WF of consumption consistently reduce, due to lower food intake, are meat, cereals and added sugar. Product groups that consistently increase in WF of consumption are fruit as well as vegetables, as the MEDIT diet recommends a high intake in these products. Also the WF of fish and seafood increases consistently (apart from Spain).

**Table 3:** WF decrease (arrow down green box), increase (arrow up orange box) or constant (arrow right yellow box) per product group, when shifting from the REF diet to the MEDIT and EAT-LANCET diet

Within the Maghreb countries as well as Egypt, the total MEDIT WF is quite similar to the total REF WF (Morocco -4%, Algeria +5%, Tunisia +2% and Egypt -4%). However, the WF amounts and proportions of certain product groups change drastically. The group “cereals and sugar” show a consistent large decrease in WF whereas crop oils, fruit and vegetables show a consistent large increase in the Maghreb countries and Egypt.

The EAT-LANCET diet reduces the WF as compared to the REF diet for all nations consistently, within the range -17% (Algeria) to -48% (Italy). In the European countries and Turkey the reductions are the largest (range -38% to -48%). In Egypt and the Maghreb countries they are the lowest (-17% to -28%). For all countries, consistent WF reductions are observed for meat, sugar and cereals (Table 3).

The EAT LANCET diet proves to require less water resources than the MEDIT diet. The MEDIT diet recommends a higher intake in meat, fruit, vegetables and milk (products) as compared to the EAT-LANCET diet. The latter recommends a higher intake in cereals, pulses, nuts and oilcrops and allows more additional sugar intake. Consequently, the largest WF proportions for MEDIT are made up by meat, crop oils, milk and fruit (Table 4). For EAT-LANCET the largest WF proportions are made up by meat, cereals and pulses, nuts and oilcrops.

**Table 4:** Heat map of the proportion (%) of the WF of different food groups to the total WF for MEDIT and EAT-LANCET. The product groups stimulants, spices and alcoholic beverages are not taken into account. (Dark red highest value to dark green lowest value). Values per country as well as an overall population-weighted average for all nine countries

**Fig. 4:**
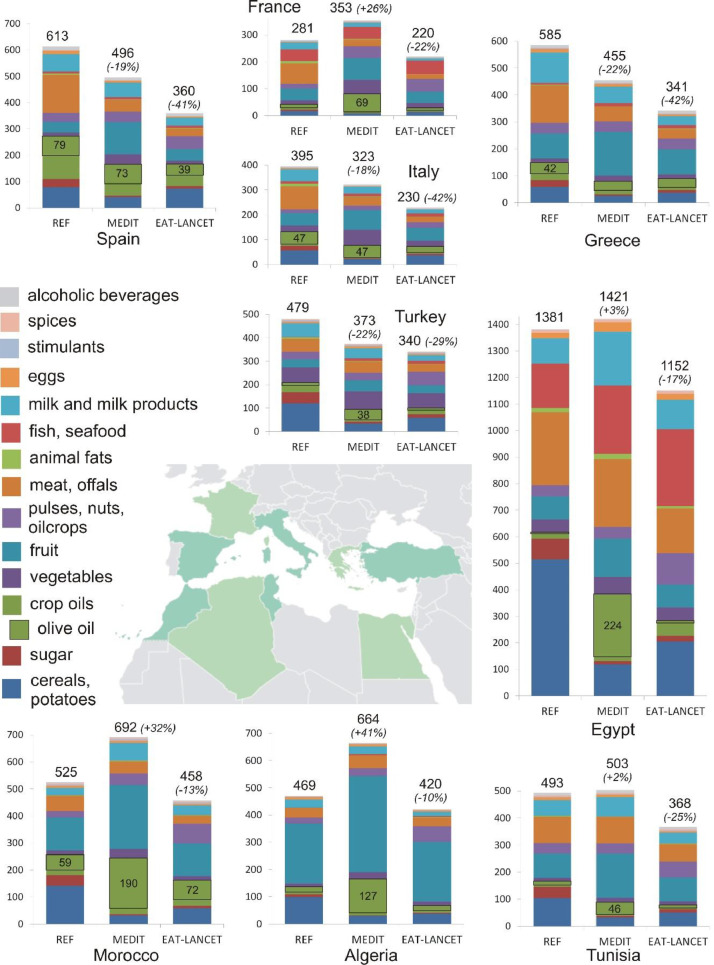
The blue WF of consumption (in litres per person per day or l/person/day) for the nine countries, for REF, MEDIT and EAT-LANCET.

When only blue water is assessed, some observations differ (Fig. 4). Total REF, MEDIT and EAT-LANCET blue WF amounts are much smaller than the green and blue WF amounts (Fig. 3). This shows the importance of green water for food security in these countries. Egypt, where the blue WF makes up about half of the green and blue WF, is a special case, as the country is highly dependent on blue water from the Nile for its food supply.

For MEDIT, the change in blue WF with respect to REF has a wide range from +41% (Algeria) to -22% (Greece and Turkey). In the European countries (except France) and Turkey a reduction is observed (-18% to -22%), whereas in Egypt and the Maghreb countries an increase in observed (+2% to +41%). The increase in blue WF in these countries and France is to a large extent accountable to an increase in blue WF for the product groups crop oils and fruit. For crop oils, particularly the recommended higher intake in olive oil increases the blue WF substantially, from 59 to 190 l/person/day in Morocco, 19 to 127 l/person/day in Algeria, 14 to 46 l/person/day in Tunisia, 2 to 224 l/person/day in Egypt and 12 to 69 l/person/day in France. That is because olive oil has a higher total and blue WF per unit than many other oils, and shifting to a higher olive oil consumption increases the total (blue) WF of oils within these countries. Within the other European countries and Turkey, the WF of olive oil does not change a lot between REF and MEDIT (such as Spain from 79 to 73 l/person/day and Italy 47 to 47 l/person/day), as the per capita intake is close to recommended amounts. Fruit also accounts for a substantial increase in the total blue WF, as much of the fruit produced in the Mediterranean region is irrigated.

For EAT-LANCET, the blue WF decreases with respect to REF consistently for all countries (range -10% for Algeria to -42% for Turkey and Greece).

As is the case for the green plus blue WF, the EAT LANCET diet proves to require less blue water resources than the MEDIT diet. The largest blue WF proportions for MEDIT are made up by fruit and crop oils (Table 5). For EAT-LANCET these are fruit, cereals and pulses, nuts and oilcrops. This observation is consistent with the review of Harris et al. (2019), who found that cereals, fruits, nuts, and oils are major contributors to the blue WF of diets.

**Table 5:** Heat map of the proportion (%) of the BLUE WF of different food groups to the total WF for MEDIT and EAT-LANCET. The product groups stimulants, spices and alcoholic beverages are not taken into account. (Dark red highest value to dark green lowest value). Values per country as well as an overall population-weighted average for all nine countries

## 4 Discussion

### 4.1 General

Countries can take additional measures to reduce the WF of their food consumption. This includes the sustainable intensification of food production employing specific WF benchmarks (Mekonnen and Hoekstra, 2014), choice in consumption of specific products with lower WFs within a food product group (Vanham et al., 2020) or the reduction of food losses and waste along the food supply chain including by consumers (Kummu et al., 2012).

We find that olive oil and fruit have large proportions in the total REF, MEDIT and EAT-LANCET WF (Tables 4 and 5). In the Maghreb countries, the green plus blue WF of production (m^3^/ton) of these products is generally much higher as compared to the global average (Fig. 2). In many regions of the Maghreb countries, also the blue WF of production is much higher than the global average. These values, in addition to a high product intake, explain the high green plus blue as well as blue WF of consumption for olive oil and fruit in the Maghreb countries (Figs. 3 and 4). Reducing the unit WF of production of these products in the Maghreb countries to WF benchmarks (Mekonnen and Hoekstra, 2014), by means of sustainable intensification including integrated water and land management (Mueller et al., 2012; Willett et al., 2019), is thereby an additional measure to decrease the WF of consumption. European countries (Greece, Italy, Spain) and Turkey show generally lower green plus blue and blue unit WFs as compared to the global average. The potential in saving water by attaining a benchmark is therefore lower, although locally each production system should be evaluated on its efficiency.

**Fig. 5:**
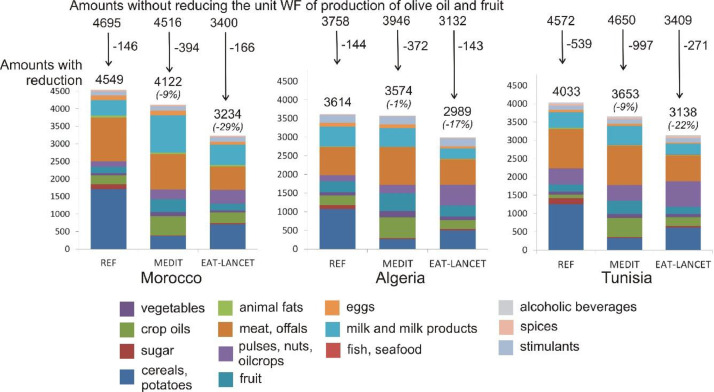
The green plus blue WF of consumption (in l/person/day) for the Maghreb countries, for REF, MEDIT and EAT-LANCET, with additionally reducing the WF of production (m^3^/ton) of olive oil and the main fruit products (oranges, apples, grapes, dates) to the global average.

We chose to reduce unit WF of production amounts of olive oil and the most consumed fruits (oranges, apples, grapes and dates) in the Maghreb countries to the global average. Such interventions reduce the WF of consumption of REF, MEDIT and EAT-LANCET consistently in all Maghreb countries, within the range 144 to 997 l/person/day (Fig. 5). Especially for the MEDIT diet - with high olive oil and fruit intake - these water efficiency measures reduce the WF substantially (by -394 l/person/day in Morocco, -372 l/person/day in Algeria and -997 l/person/day in Tunisia). These measures also result in a consistent reduction of the MEDIT and EAT-LANCET WF with respect to the REF WEF (ranges -1% to -9% respectively -17% to -29%), whereas without implementing them this was not the case for Algeria and Tunisia (Fig. 3). Again, the EAT-LANCET diet proves to require less water than the MEDIT diet.

Another option in further reducing the WF of consumption of food product groups such as crop oils is to critically assess which oils are consumed within a country. Different crop oils required different amounts of water to produce (Fig. 1b). Olive oil proves to have a high green plus blue as well as blue unit WF of production (14504 respectively 2437 m^3^/ton), as compared to sunflower oil (6387 respectively 299 m^3^/ton), rapeseed oil (14504 respectively 2437 m^3^/ton) or soybean oil (14504 respectively 2437 m^3^/ton). Nations such as the Maghreb countries can, based on their available water resources, strategically promote consumption of less water-demanding oils and/or choose to increasingly import water-demanding oils such as olive oil. In such countries, the nutritional recommendation of olive oil intake of the MEDIT diet (half of fats from olive oil) could be revised according to their local water availability. Latter considerations can be extended to other food groups.

A full sustainability assessment of a dietary WF encompasses different components (Vanham, 2020): (1) an equity assessment of the total WF amount; (2) an efficiency assessment for each food item in the diet as well as (3) an impact assessment (blue water stress and green water scarcity) for each food item in the diet. Our study partly addresses the points 1 (equity) and 2 (efficiency), but not point 3 (impact). An equity assessment is necessary as the global pool of both blue and green environmentally available water resources is limited (Mekonnen and Hoekstra, 2016; Rockstrom et al., 2009; Schyns et al., 2019), meaning that – according to the equity principle – only a certain amount of water is globally available per person within a specific time period. In line with that principle, a lower total dietary WF is better than a higher one. In our assessment, we identify which dietary WFs are better (lower) from this perspective. Adherence to such diets would thereby contribute to SDG Target 6.4 “The reduction of global water scarcity”. The second point “efficiency”, implies the evaluation of the WF of each food product within the diet towards a benchmark (Mekonnen and Hoekstra, 2014; Vanham and Leip, 2020; Vanham and Mekonnen, 2021). We partly address this point by including efficiency scenarios for selected food items. Within the SDG framework, this relates to indicator 6.4.1 “Change in water use efficiency over time”. We do not address point 3, which implies the evaluation of the local blue and green water stress/scarcity of each food product within the diet (Mekonnen and Hoekstra, 2020; Vanham and Leip, 2020). Impact should be low, otherwise the product is considered unsustainable. For blue water stress, the related SDG indicator is indicator 6.4.2 “Level of water stress” (Vanham et al., 2018b).

Whether a healthy dietary pattern is sustainable, requires the analysis of many different environmental, economic as well as sociocultural indicators (FAO and WHO, 2019; Hachem et al., 2020). Such additional indicators include the affordability of a diet (Hirvonen et al., 2020) or farmer income. Here we address the specific environmental aspect of water quantity by means of the WF concept. It is clear that integrated policy options such as the EU Farm to Fork Strategy (EC, 2020) need to be based on a comprehensive indicator set and not on just one indicator. Trade-offs and win-win options need to be identified. As an example, nuts are a nutritional good choice and treenuts can perform well on greenhouse gas emissions, but they have very high unit WF amounts and in the Mediterranean region, large quantities are produced under blue water stress (Vanham et al., 2020). Also, our assessment shows that from a water perspective, Maghreb countries might want to shift olive oil production and/or consumption to other vegetal oils, but this can from a nutritional, economic or socio-cultural point of view not be the best option. As an example, olive oil production and olive groves have a long historical tradition in the Mediterranean region, are an integral part of the (rural) landscape, are important for agricultural income and employment and have value in agritourism (Loumou and Giourga, 2003; Moreira et al., 2019; Pulido-Fernández et al., 2019; Salmoral et al., 2011; Torres-Miralles et al., 2017). Also, the water-efficiency measures we describe for olive oil and fruits, should be implemented in a sustainable manner, respecting biodiversity and the ecosystem services provided by healthy agricultural landscapes (Moreira et al., 2019), which shows the importance of additional environmental indicators on pollution and biodiversity (Hachem et al., 2020). Therefore, our analysis needs to be seen in the light of a bigger framework addressing multiple indicators.

### 4.2 Data quality and limitations

We use FAO FBS data as well as existing WF data (Mekonnen and Hoekstra, 2012; Mekonnen and Hoekstra, 2011). Latter data represent average values for the period 1996-2005 and are the highest quality data that exist on food product WFs. FAO FBS have certain limitations, but they provide a cost-efficient and effective database of assessing longitudinal comparisons of dietary patterns within and between nations (Vilarnau et al., 2019). These data tend to over-estimate consumption (Del Gobbo et al., 2015), which we partly compensate by working with conversion factors as described in the methodology. More detailed assessments using national dietary surveys would provide additional WF results which should be compared with the results we present here (Vanham, 2020). We thus only provide information on national averages that can be used for national policy guidance. Using national dietary surveys also provides the possibility to quantify differences in WFs for different socio-economic classes or geographical regions within a country. Such assessments have been addressed in other studies, e.g. Vanham et al. (2018a), Harris et al. (2017) or Koteswara Rao and Chandrasekharam (2019).

## 5 Conclusions

The diet scenarios we assess in our paper are important parts of the solution to obtain the sustainable use of water resources in Mediterranean countries. The EAT-Lancet universal reference and the Mediterranean diet have been identified as relatively similar diets, as both are low in the intake of animal products such as meat and milk and high in the intake of fruit, vegetables, pulses, nuts and oilcrops. We find that the EAT-Lancet diet requires less water than the Mediterranean diet. This is because the MEDIT recommends a higher intake of meat, milk, olive oil and fruit as compared to EAT-LANCET, whereas EAT-LANCET recommends a higher intake in cereals, as well as pulses, nuts and oilcrops. This does imply that EAT-LANCET could be more difficult to achieve as the change in meat consumption is larger than for MEDIT. Omnivores are generally unwilling to change their meat consumption (Valli et al., 2019). In addition, current adherence to MEDIT in the countries of the Mediterranean (Hachem et al., 2020) will probably be larger than adherence to EAT-LANCET, making shifts to MEDIT more likely due to the social norm effect (Eker et al., 2019).

Total WFs for REF, MEDIT and EAT-LANCET differ between countries, due to local climatological conditions, agricultural practices, the rate of import of products as well as cultural preferences in food consumption. Apart from these dietary shifts, sustainable water resource use needs also other interventions, such as resource efficiency in water use (attaining WF benchmarks) or the preferred consumption of less water-demanding products within a food product group (such as partly substituting olive oil with sunflower or rapeseed oil). Critically evaluating and implementing such options is especially needed in the countries of the southern Mediterranean basin, where large population increases are projected. In the Maghreb countries, increasing the water efficiency of olive oil and fruit production would substantially reduce the total dietary WF.

These interventions can contribute to achieve SDG Target 6.4, which aims at reducing global water scarcity. Integrated food system policies can find valuable information in our WF analysis, by identifying trade-offs and win-win options with other environmental, economic and sociocultural indicators.

## CREDIT Author Statement

**Davy Vanham:** Conceptualization, Data analysis, Visualization, Writing- Original draft preparation, **Susann Guenther**: Data preparation, data analysis **Marta Ros**-**Baró:** Data analysis, Writing- Reviewing and Editing **Anna Bach-Faig:** Conceptualization, Data analysis, Writing- Reviewing and Editing

## Declaration of Competing Interest

The authors declare that they have no known competing financial interests or personal relationships that could have appeared to influence the work reported in this paper.

## Appendix Supplementary materials

Image, application 1
