# Supplementary material for: Which diet has the lower water footprint in Mediterranean countries?
Source: Resour Conserv Recycl. 2021 Aug;171:105631. doi: 10.1016/j.resconrec.2021.105631 (PMC8216694; doi:10.1016/j.resconrec.2021.105631)
Supplement: Supplementary file 1 [file mmc1.docx]

**Supplementary table 1:** National WF of consumption (l/person/day) per product group for REF, MEDIT and EAT-LANCET

| **Product group** | **REF** | | | **MEDIT** | | | **EAT-LANCET** | | |
| --- | --- | --- | --- | --- | --- | --- | --- | --- | --- |
|  | **green** | **blue** | **total** | **green** | **blue** | **total** | **green** | **blue** | **total** |
| **European countries: Spain** | | | | | | | | | |
| cereals, potatoes | 373 | 79 | 451 | 193 | 41 | 233 | 363 | 73 | 435 |
| sugar | 60 | 31 | 91 | 11 | 5 | 16 | 19 | 10 | 29 |
| crop oils | 566 | 163 | 729 | 451 | 119 | 570 | 289 | 83 | 372 |
| vegetables | 22 | 13 | 36 | 64 | 38 | 102 | 22 | 13 | 36 |
| fruit | 68 | 41 | 109 | 204 | 123 | 326 | 75 | 45 | 120 |
| Pulses, nuts, oilcrops | 224 | 34 | 258 | 260 | 39 | 299 | 344 | 49 | 393 |
| Meat, offals | 1240 | 144 | 1385 | 389 | 45 | 434 | 258 | 30 | 288 |
| animal fats | 45 | 6 | 50 | 14 | 2 | 16 | 15 | 2 | 17 |
| fish and seafood | 25 | 8 | 33 | 25 | 8 | 33 | 23 | 7 | 31 |
| Milk, milk products | 503 | 65 | 569 | 427 | 56 | 482 | 235 | 31 | 265 |
| eggs | 104 | 13 | 117 | 55 | 7 | 62 | 33 | 4 | 37 |
| stimulants | 285 | 1 | 287 | 285 | 1 | 287 | 285 | 1 | 287 |
| spices | 5 | 1 | 6 | 5 | 1 | 6 | 5 | 1 | 6 |
| alcoholic beverages | 103 | 14 | 118 | 86 | 12 | 98 | 86 | 12 | 98 |
| **total** | **3625** | **613** | **4238** | **2469** | **496** | **2966** | **2054** | **360** | **2414** |
| **Reduction (%)** |  |  |  | **-32** | **-19** | **-30** | **-43** | **-41** | **-43** |
| **European countries: France** | | | | | | | | | |
| cereals, potatoes | 234 | 17 | 251 | 105 | 8 | 112 | 193 | 13 | 206 |
| sugar | 35 | 5 | 39 | 5 | 1 | 6 | 9 | 1 | 10 |
| crop oils | 288 | 21 | 309 | 413 | 71 | 483 | 203 | 15 | 218 |
| vegetables | 44 | 15 | 59 | 159 | 54 | 213 | 53 | 18 | 71 |
| fruit | 175 | 43 | 218 | 332 | 81 | 413 | 175 | 43 | 218 |
| Pulses, nuts, oilcrops | 98 | 18 | 117 | 230 | 43 | 273 | 268 | 46 | 314 |
| Meat, offals | 1246 | 76 | 1321 | 412 | 25 | 437 | 275 | 17 | 292 |
| animal fats | 97 | 8 | 105 | 30 | 3 | 33 | 11 | 1 | 12 |
| fish and seafood | 13 | 44 | 57 | 14 | 45 | 59 | 15 | 50 | 66 |
| Milk, milk products | 332 | 25 | 357 | 197 | 15 | 212 | 108 | 8 | 116 |
| eggs | 49 | 3 | 52 | 27 | 2 | 29 | 17 | 1 | 18 |
| stimulants | 458 | 3 | 461 | 458 | 3 | 461 | 458 | 3 | 461 |
| spices | 6 | 1 | 6 | 6 | 1 | 6 | 6 | 1 | 6 |
| alcoholic beverages | 90 | 3 | 93 | 70 | 3 | 73 | 70 | 3 | 73 |
| **total** | **3165** | **281** | **3446** | **2457** | **353** | **2810** | **1861** | **220** | **2080** |
| **Reduction (%)** |  |  |  | **-22** | **+26** | **-18** | **-41** | **-22** | **-40** |
| **European countries: Italy** | | | | | | | | | |
| cereals, potatoes | 506 | 57 | 563 | 186 | 22 | 208 | 343 | 38 | 381 |
| sugar | 45 | 18 | 63 | 8 | 3 | 11 | 14 | 6 | 20 |
| crop oils | 478 | 56 | 534 | 368 | 51 | 419 | 243 | 29 | 271 |
| vegetables | 51 | 25 | 76 | 133 | 64 | 197 | 51 | 25 | 76 |
| fruit | 227 | 51 | 279 | 349 | 79 | 427 | 227 | 51 | 279 |
| Pulses, nuts, oilcrops | 107 | 15 | 122 | 136 | 19 | 155 | 188 | 22 | 210 |
| Meat, offals | 1204 | 92 | 1296 | 431 | 33 | 464 | 266 | 20 | 286 |
| animal fats | 108 | 11 | 119 | 28 | 3 | 31 | 14 | 1 | 16 |
| fish and seafood | 4 | 9 | 12 | 5 | 12 | 17 | 5 | 13 | 19 |
| Milk, milk products | 462 | 49 | 512 | 259 | 28 | 286 | 142 | 15 | 158 |
| eggs | 42 | 4 | 47 | 24 | 2 | 26 | 15 | 1 | 16 |
| stimulants | 261 | 2 | 263 | 261 | 2 | 263 | 261 | 2 | 263 |
| spices | 2 | 0 | 2 | 2 | 0 | 2 | 2 | 0 | 2 |
| alcoholic beverages | 60 | 5 | 65 | 60 | 5 | 65 | 60 | 5 | 65 |
| **total** | **3557** | **395** | **3952** | **2248** | **323** | **2571** | **1830** | **230** | **2060** |
| **Reduction (%)** |  |  |  | **-37** | **-18** | **-35** | **-49** | **-42** | **-48** |
| **European countries: Greece** | | | | | | | | | |
| cereals, potatoes | 534 | 58 | 593 | 221 | 25 | 246 | 418 | 38 | 455 |
| sugar | 61 | 25 | 87 | 12 | 5 | 17 | 22 | 9 | 31 |
| crop oils | 377 | 66 | 443 | 275 | 48 | 323 | 252 | 44 | 296 |
| vegetables | 32 | 14 | 46 | 49 | 21 | 71 | 32 | 14 | 46 |
| fruit | 158 | 94 | 252 | 276 | 163 | 439 | 158 | 94 | 252 |
| Pulses, nuts, oilcrops | 214 | 40 | 254 | 214 | 40 | 254 | 214 | 40 | 254 |
| Meat, offals | 1218 | 139 | 1357 | 486 | 55 | 541 | 305 | 36 | 341 |
| animal fats | 28 | 3 | 31 | 9 | 1 | 10 | 19 | 2 | 21 |
| fish and seafood | 31 | 6 | 37 | 58 | 11 | 69 | 64 | 12 | 76 |
| Milk, milk products | 635 | 113 | 749 | 342 | 61 | 404 | 188 | 34 | 222 |
| eggs | 67 | 13 | 81 | 52 | 10 | 62 | 32 | 6 | 38 |
| stimulants | 372 | 3 | 375 | 372 | 3 | 375 | 372 | 3 | 375 |
| spices | 9 | 1 | 10 | 9 | 1 | 10 | 9 | 1 | 10 |
| alcoholic beverages | 43 | 10 | 53 | 43 | 10 | 53 | 43 | 10 | 53 |
| **total** | **3782** | **585** | **4367** | **2419** | **455** | **2874** | **2128** | **341** | **2470** |
| **Reduction (%)** |  |  |  | **-36** | **-22** | **-34** | **-44** | **-42** | **-43** |
| **Eastern Mediterranean countries: Turkey** | | | | | | | | | |
| cereals, potatoes | 1029 | 120 | 1148 | 268 | 34 | 302 | 516 | 58 | 574 |
| sugar | 35 | 48 | 82 | 6 | 8 | 14 | 11 | 15 | 26 |
| crop oils | 287 | 41 | 328 | 406 | 53 | 459 | 176 | 25 | 202 |
| vegetables | 59 | 65 | 124 | 70 | 77 | 147 | 59 | 65 | 124 |
| fruit | 127 | 34 | 161 | 174 | 47 | 221 | 127 | 34 | 161 |
| Pulses, nuts, oilcrops | 194 | 33 | 227 | 194 | 33 | 227 | 234 | 57 | 292 |
| Meat, offals | 921 | 56 | 977 | 779 | 47 | 826 | 516 | 31 | 547 |
| animal fats | 44 | 5 | 49 | 17 | 2 | 19 | 21 | 3 | 24 |
| fish and seafood | 3 | 2 | 5 | 15 | 12 | 27 | 16 | 13 | 30 |
| Milk, milk products | 456 | 58 | 514 | 340 | 43 | 383 | 187 | 24 | 211 |
| eggs | 71 | 7 | 79 | 68 | 7 | 75 | 41 | 4 | 45 |
| stimulants | 63 | 6 | 69 | 63 | 6 | 69 | 63 | 6 | 69 |
| spices | 34 | 4 | 38 | 34 | 4 | 38 | 34 | 4 | 38 |
| alcoholic beverages | 11 | 0 | 11 | 11 | 0 | 11 | 11 | 0 | 11 |
| **total** | **3333** | **479** | **3812** | **2446** | **373** | **2819** | **2013** | **340** | **2353** |
| **Reduction (%)** |  |  |  | **-27** | **-22** | **-26** | **-40** | **-29** | **-38** |
| **Eastern Mediterranean countries: Egypt** | | | | | | | | | |
| cereals, potatoes | 498 | 515 | 1012 | 104 | 119 | 223 | 200 | 206 | 406 |
| sugar | 37 | 77 | 115 | 7 | 12 | 18 | 12 | 21 | 33 |
| crop oils | 57 | 25 | 82 | 116 | 252 | 368 | 133 | 58 | 191 |
| vegetables | 68 | 48 | 116 | 93 | 66 | 160 | 68 | 48 | 116 |
| fruit | 89 | 86 | 176 | 150 | 144 | 294 | 89 | 86 | 176 |
| Pulses, nuts, oilcrops | 38 | 43 | 81 | 42 | 45 | 87 | 123 | 118 | 241 |
| Meat, offals | 514 | 275 | 790 | 477 | 255 | 732 | 316 | 169 | 485 |
| animal fats | 24 | 16 | 39 | 29 | 19 | 48 | 14 | 10 | 24 |
| fish and seafood | 51 | 168 | 219 | 78 | 258 | 337 | 88 | 290 | 378 |
| Milk, milk products | 100 | 95 | 194 | 213 | 202 | 415 | 117 | 111 | 228 |
| eggs | 18 | 20 | 38 | 31 | 35 | 66 | 19 | 21 | 40 |
| stimulants | 44 | 1 | 45 | 44 | 1 | 45 | 44 | 1 | 45 |
| spices | 14 | 12 | 25 | 14 | 12 | 25 | 14 | 12 | 25 |
| alcoholic beverages | 0 | 1 | 2 | 0 | 1 | 2 | 0 | 1 | 2 |
| **total** | **1552** | **1381** | **2933** | **1398** | **1421** | **2819** | **1237** | **1152** | **2389** |
| **Reduction (%)** |  |  |  | **-10** | **+3** | **-4** | **-20** | **-17** | **-19** |
| **Maghreb countries: Tunisia** | | | | | | | | | |
| cereals, potatoes | 1148 | 104 | 1252 | 294 | 32 | 326 | 562 | 51 | 613 |
| sugar | 126 | 42 | 167 | 19 | 6 | 25 | 35 | 11 | 46 |
| crop oils | 546 | 19 | 565 | 1354 | 49 | 1403 | 426 | 15 | 441 |
| vegetables | 71 | 15 | 86 | 86 | 18 | 104 | 71 | 15 | 86 |
| fruit | 175 | 89 | 264 | 322 | 163 | 485 | 175 | 89 | 264 |
| Pulses, nuts, oilcrops | 398 | 39 | 437 | 390 | 38 | 428 | 640 | 58 | 698 |
| Meat, offals | 983 | 97 | 1080 | 980 | 97 | 1077 | 649 | 64 | 713 |
| animal fats | 23 | 3 | 26 | 12 | 2 | 14 | 18 | 3 | 20 |
| fish and seafood | 2 | 0 | 2 | 4 | 0 | 4 | 4 | 0 | 5 |
| Milk, milk products | 372 | 58 | 430 | 454 | 71 | 525 | 250 | 39 | 289 |
| eggs | 54 | 10 | 63 | 51 | 9 | 60 | 31 | 6 | 36 |
| stimulants | 115 | 3 | 118 | 115 | 3 | 118 | 115 | 3 | 118 |
| spices | 42 | 5 | 47 | 42 | 5 | 47 | 42 | 5 | 47 |
| alcoholic beverages | 24 | 10 | 34 | 24 | 10 | 34 | 24 | 10 | 34 |
| **total** | **4079** | **493** | **4572** | **4147** | **503** | **4650** | **3041** | **368** | **3409** |
| **Reduction (%)** |  |  |  | **+2** | **+2** | **+2** | **-25** | **-25** | **-25** |
| **Maghreb countries: Algeria** | | | | | | | | | |
| cereals, potatoes | 973 | 99 | 1071 | 241 | 30 | 271 | 463 | 39 | 501 |
| sugar | 97 | 10 | 107 | 18 | 2 | 20 | 33 | 3 | 36 |
| crop oils | 253 | 27 | 280 | 615 | 134 | 749 | 244 | 26 | 270 |
| vegetables | 76 | 14 | 90 | 141 | 25 | 166 | 76 | 14 | 90 |
| fruit | 194 | 220 | 414 | 311 | 352 | 663 | 194 | 220 | 414 |
| Pulses, nuts, oilcrops | 145 | 21 | 166 | 195 | 28 | 223 | 499 | 58 | 557 |
| Meat, offals | 718 | 36 | 754 | 968 | 48 | 1017 | 641 | 32 | 673 |
| animal fats | 9 | 1 | 10 | 6 | 0 | 7 | 19 | 1 | 20 |
| fish and seafood | 0 | 0 | 0 | 1 | 3 | 4 | 1 | 4 | 5 |
| Milk, milk products | 503 | 30 | 533 | 468 | 28 | 497 | 258 | 16 | 273 |
| eggs | 88 | 9 | 97 | 86 | 8 | 95 | 52 | 5 | 57 |
| stimulants | 211 | 1 | 212 | 211 | 1 | 212 | 211 | 1 | 212 |
| spices | 14 | 2 | 16 | 14 | 2 | 16 | 14 | 2 | 16 |
| alcoholic beverages | 7 | 0 | 7 | 7 | 0 | 7 | 7 | 0 | 7 |
| **total** | **3289** | **469** | **3758** | **3282** | **664** | **3946** | **2712** | **420** | **3132** |
| **Reduction (%)** |  |  |  | **0** | **+41** | **+5** | **-18** | **-10** | **-17** |
| **Maghreb countries: Morocco** | | | | | | | | | |
| cereals, potatoes | 1569 | 142 | 1711 | 339 | 32 | 371 | 647 | 58 | 705 |
| sugar | 99 | 39 | 137 | 13 | 5 | 19 | 24 | 9 | 34 |
| crop oils | 261 | 76 | 337 | 615 | 208 | 823 | 321 | 94 | 415 |
| vegetables | 42 | 16 | 58 | 87 | 33 | 120 | 42 | 16 | 58 |
| fruit | 126 | 121 | 247 | 248 | 236 | 484 | 126 | 121 | 247 |
| Pulses, nuts, oilcrops | 134 | 25 | 159 | 235 | 43 | 278 | 322 | 73 | 395 |
| Meat, offals | 1191 | 55 | 1245 | 963 | 44 | 1008 | 638 | 29 | 667 |
| animal fats | 53 | 4 | 57 | 40 | 3 | 43 | 35 | 2 | 37 |
| fish and seafood | 0 | 1 | 1 | 1 | 1 | 2 | 1 | 1 | 2 |
| Milk, milk products | 415 | 27 | 442 | 993 | 65 | 1058 | 546 | 36 | 582 |
| eggs | 118 | 8 | 126 | 128 | 8 | 137 | 78 | 5 | 83 |
| stimulants | 120 | 4 | 125 | 120 | 4 | 125 | 120 | 4 | 125 |
| spices | 35 | 5 | 39 | 35 | 5 | 39 | 35 | 5 | 39 |
| alcoholic beverages | 7 | 4 | 11 | 7 | 4 | 11 | 7 | 4 | 11 |
| **total** | **4171** | **525** | **4695** | **3825** | **692** | **4516** | **2942** | **458** | **3400** |
| **Reduction (%)** |  |  |  | **-8** | **+32** | **-4** | **-29** | **-13** | **-28** |
